# MDCT-based Grading of Perirenal Changes Secondary to Acute Unilateral Upper Urinary Tract Obstruction

**DOI:** 10.2174/0115734056329112250710123850

**Published:** 2025-07-22

**Authors:** Fukang Zhang, Huayu You, Yanlan Deng, Guiquan Chen, Yihui Qiu, Zhiyong Ling, Huasong Cai, Nan Liu

**Affiliations:** 1 Department of Radiology, The Seventh Affiliated Hospital, Southern Medical University, Foshan 528244, PR China; 2 Department of Radiology, The First Affiliated Hospital, Sun Yat-Sen University, Guangzhou, PR China; 3 Institute of Environment and Health, Pinghu Hospital, Shenzhen University, Shenzhen, China; £ Present Address: Institute of Environment and Health, South China Hospital, Medical School, Shenzhen University, Shenzhen 518116, PR China

**Keywords:** Acute unilateral upper urinary tract obstruction (AUUTO), Multidetector spiral computed tomography, Perinephric space, Perinephric bridging septa, Hydronephrosis, Infection, GFR

## Abstract

**Background:**

Unilateral upper ureteral obstruction is one of the most common causes of acute kidney function impairment. Grading perirenal changes secondary to acute unilateral upper urinary tract obstruction (AUUTO) with multidetector spiral computed tomography (MDCT) and exploring its association with kidney function are useful for diagnosing and assessing damage to the ipsilateral kidney. However, the correlation between renal function impairment and the severity of perinephric changes secondary to AUUTO has not been reported.

**Objective:**

This study aimed to investigate the association of perirenal changes secondary to AUUTO with hydronephrosis and serum creatinine levels, as well as white blood cell counts.

**Methods:**

This retrospective study included 376 patients with acute unilateral upper ureteral obstruction, all of whom were subjected to MDCT scans. They were classified into four grades (0-III) according to their perirenal changes on MDCT images. The severity of hydronephrosis was classified into four grades based on MDCT scans. The serum creatinine level and leukocyte counts were compared among the MDCT grade groups, and logistic regression analysis was conducted.

**Results:**

Among 376 patients, 77 (20.5%), 103 (27.4%), 140 (37.2%), and 56 (14.9%) cases were graded into MDCT 0, I, II, and III, respectively. The proportions of patients who had normal kidneys in MDCT 0, I, II, and III were 20 (26.0%), 10 (9.7%), 11(7.9%), and 3 (5.4%), respectively. The proportions of patients who had mild hydronephrosis in MDCT 0, I, II, and III were 55 (71.4%), 83 (80.6%), 118 (84.2%), and 46 (82.1%), respectively. The proportions of patients who had moderate and severe hydronephrosis in MDCT 0, I, II, and III were 2(2.6%), 10 (9.7%), 11 (7.9%), 7 (12.5%), respectively. Serum creatinine levels and white blood cell counts were significantly different among the MDCT grade groups (*P* < 0.001). Univariate and multivariate logistic regression analyses indicated that the serum creatinine level and white blood cell counts were positively associated with the MDCT grades (*P* < 0.001).

**Conclusion:**

Perinephric changes secondary to AUUTO on MDCT images were associated with the degree of obstruction. The severity of perinephric changes can reflect the functional impairment in the ipsilateral kidney. The MDCT grades may aid clinicians in assessing renal function impairment early in patients with AUUTO, which may help patients receive early intervention and avoid the potential risk of infection and deterioration of renal function.

## 1 INTRODUCTION

Unilateral upper urinary tract obstruction is one of the most common causes of acute renal function impairment. Urinary stone, an important aetiology of acute ureteral obstruction, has a prevalence of 5.7% in China [1]. The anomalous changes in the kidney and the perinephric space caused by (AUUTO) are universally considered renal physiological adaptations and responses of the kidney. Urinary tract obstruction blocks the urine outflow from the kidney, leading to an increase in pressure in collecting system, pathological distension of the renal pelvis, and hydronephrosis. If left untreated, it would develop into progressive renal atrophy and impair the kidney’s function, ultimately resulting in kidney failure [2].

The method for evaluating acute kidney function decline is an important topic in clinical research. The glomerular filtration rate (GFR) is a sensitive marker reflecting renal function [3]. Radiopharmaceuticals such as ^99m^Tc-DPTA can be used for detecting the glomerular filtration rate, thereby evaluating the renal function. However, renal scans are expensive, and most general hospitals lack equipment for this examination, so they cannot meet the needs of clinics [4]. At the same time, radiopharmaceuticals may further induce renal function impairment, as they are primarily cleared through glomerular filtration. Their reabsorption and retention in the proximal renal tubules expose the kidneys to additional radiation [5, 6]. Therefore, this test is not the first recommendation for patients with acute urinary tract obstruction.

Acute upper urinary tract obstruction is often associated with the elevation of serum creatinine (Cr) [7, 8]. Serum Cr levels are widely used for evaluating renal function [4, 9]. However, serum Cr levels reflect the overall function of bilateral kidneys [4, 9]. Renal function may have declined in some patients with unilateral ureteral obstruction, and their serum Cr levels are still within the normal range because of the compensatory effect of the unaffected side [2]. It is necessary to find an effective and non-invasive method for evaluating unilateral kidney function impairment in patients with acute ureteral obstruction.

The elevation of white blood cell (WBC) is a response to inflammation, trauma or infection [10]. A clinical study showed that WBC counts significantly elevated in patients with acute kidney injury compared with healthy controls [11]. Similarly, a recent study demonstrated that elevated WBC count was a predictor of the odds of kidney function decline [12]. These studies suggested that elevated WBC counts in patients with ureteral calculi may be related to kidney function impairment.

Acute upper urinary tract obstruction may lead to secondary signs, such as hydronephrosis, perinephric fat stranding, thickening of the bridging septa and perinephric fascia, and fluid accumulation in the perinephric space in the ipsilateral kidney. The severity of hydronephrosis depends on the grade, duration, and location of the obstruction [13]. The thickening of the perinephric bridging septa was supposed to be caused by pyelosinus, pyelotubular, pyelolymphatic, and pyelovenous reflux [9]. Farrell’s group found that the perinephric fat stranding was associated with a decline in renal function [9]. However, the correlation between renal function impairment and the severity of the perinephric changes secondary to AUUTO has not been investigated.

Image technique plays an important role in the diagnosis of ureterolithiasis. Unenhanced helical CT is one of the important methods to diagnose urinary stones [2, 14]. Multidetector spiral computed tomography (MDCT) is an ideal method for diagnosing ureterolithiasis because the locations of the calculi and the changes in the kidneys and fats around the perinephric space can be easily visualized with MDCT [15-17].

This study determined the grades of perinephric changes secondary to AUUTO based on MDCT scans and investigated the relationship between hydronephrosis and MDCT grades. At the same time, this study also explored the changes in the serum Cr levels and WBC counts in different MDCT grade groups and investigated their correlations with the MDCT grades, which may provide some clinical clues for the early diagnosis of AUUTO, avoiding the potential risk of infection and deterioration of renal function.

## 2 MATERIALS AND METHODS

### 2.1 Clinical Data Collection

After obtaining institutional medical ethics committee approval in this retrospective study, we included 376 patients with AUUTO due to calculus between Jan. 2015 and Dec. 2017 in this retrospective study. Serum Cr levels and a routine blood examination were performed within 8 h after admission. All patients underwent a CT scan of the entire urinary system. The inclusion criterion was acute unilateral urinary tract obstruction due to calculus (onset within 2 weeks). Exclusion criteria included a solitary kidney, the presence of tumours in the kidney, nephritis, tuberculosis, chronic kidney disease (including chronic renal failure), ureteral lesions or obstructions caused by other factors (such as idiopathic hydronephrosis), lesions or obstructions in the contralateral kidney or ureter, or lesions in adjacent organs (such as pancreatitis).

### 2.2 MDCT Scan Equipment and Methods

The MDCT scan was performed with a 16-slice spiral CT scanner (GE/BrightSpeed, USA). All CT scans were unenhanced. The scan region extended from the upper pole of the kidney to the upper edge of the pubis. The scanning parameters are as follows: scanning tube voltage, 120 kV; 300 mAs as the mask tube current; and 120 mA as the following tube current. The other scan parameters were 0.5 mm × 320 collimator, 512 × 512 matrices, 1.375:1; slice thickness of spiral scans, 7.5 mm; and reconstructed slice thickness, 1.25 mm. Reconstruction was performed with the soft tissue algorithm. After scanning, 1.25 mm slices of the reconstructed scans were transmitted to the workstation for multiplanar reconstruction.

### 2.3 Image Analysis

The MDCT scan images were analysed by two professional radiologists on a radiology imaging software workstation with picture archiving and communication systems 5.0 (PACS 5.0, http://www.gdpacs.com/webpage/website/ solutions/pacs5.jsp), which is widely used in Chinese hospitals (Chengdu Goldisc UESTC Multimedia Technology Co., Ltd, Chengdu, China). In cases of disagreement, consensus was reached through discussion. Changes in the urinary system and perinephric space were primarily observed on the ipsilateral versus contralateral side.

#### 2.3.1 The Location of the Calculus

The upper urinary tract was divided into two segments: (1) the renal pelvis to the upper portion of the ureter (the upper margin of the sacroiliac joint); (2) the middle and lower portion of the ureter (the upper margin of the sacroiliac joint to the bladder segment). Importantly, this segmentation was used solely for descriptive categorization; all cases of acute unilateral upper urinary tract obstruction-regardless of calculus location within the renal pelvis, or the upper, middle, or lower ureter-were included in the study.

#### 2.3.2 The Grades of Hydronephrosis

**Fig. (1):**
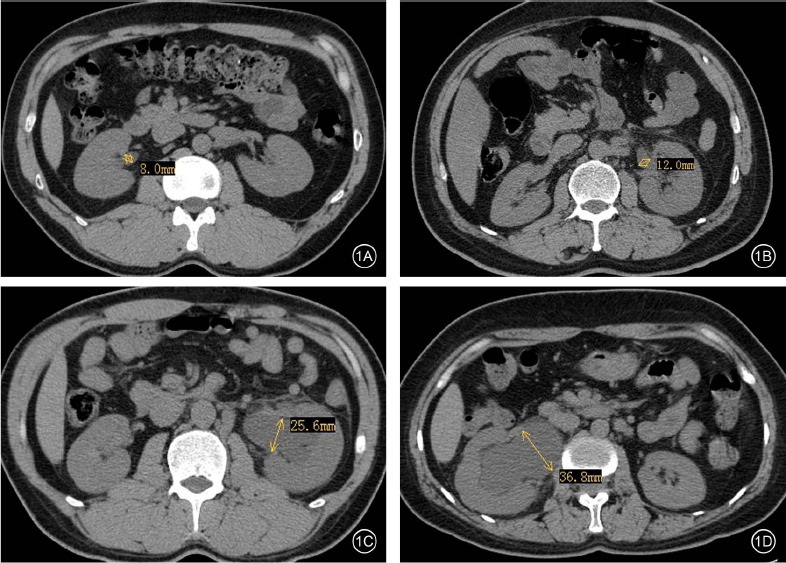
The grades of hydronephrosis in MDCT images. The representative images of 0 grade (**A**), mild grade (**B**), moderate grade (**C**) and severe grade (**D**) in the transverse MDCT image. The yellow arrow indicates the maximum renal pelvis anteroposterior diameter in different cases.

Hydronephrosis was classified into four grades according to the severity of hydronephrosis in the MDCT images as follows, using criteria modified from the previous radiology grading system [18]:

0 grade (normal): the diameter of the renal pelvis anteroposterior was below 10 mm without abnormal changes in the shape of the renal pelvis and calyces in the axial and oblique sagittal planes (Fig. **1A**).

Mild: the maximum diameter of the renal pelvis anteroposterior was between 10 and 20 mm, with minor calyces blunting (Fig. **1B**) in transverse MDCT images.

Moderate: the maximum diameter of the renal pelvis anteroposterior was between 20 and 35 mm, with minor calyces bulging and enlarging and the junction of the major calyces widening in the transverse MDCT images (Fig. **1C**).

Severe: transverse MDCT images showed that the maximum renal pelvis anteroposterior diameter was ≥ 35 mm, and the collecting system had drastic cystic-like changes accompanied by marked parenchymal thinning (Fig. **1D**).

#### 2.3.3 Grading the Perinephric changes in MDCT Images

Perinephric changes in MDCT images were evaluated on the peritoneal window, and the perinephric space was compared between the affected and contralateral sides. The manifestations of the pathological images included (1) increased fat density and thickened bridging septa in the ipsilateral perinephric space; (2) thickened ipsilateral anterior and/or posterior renal fascia; and (3) fluid accumulation in the ipsilateral perinephric space.

**Fig. (2):**
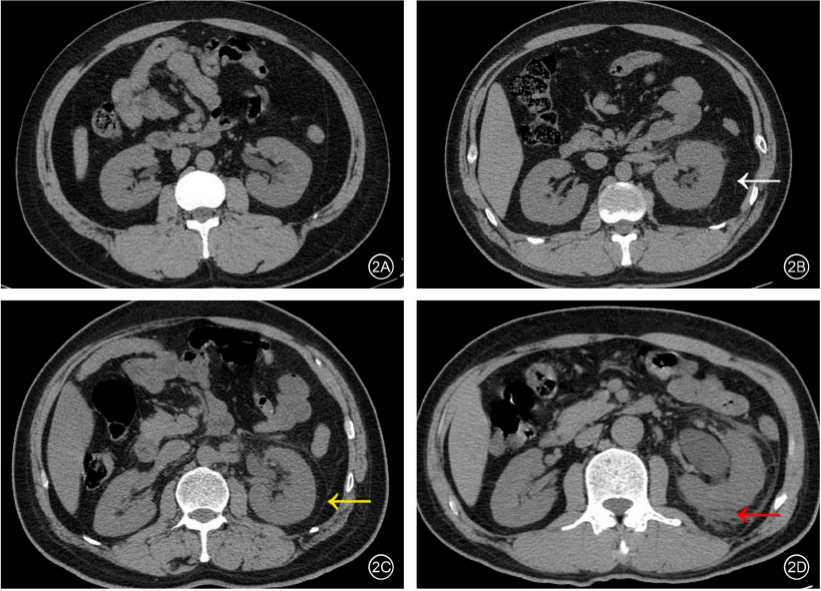
Manifestations of perinephric changes secondary to acute unilateral upper urinary tract obstruction in MDCT grade 0 (**A**), MDCT grade I (**B**), MDCT grade II (**C**), MDCT grade III (**D**). White arrow indicates fat density increased in the perinephric space, accompanied by the boundary appearing blurred with linear shadows thickening of the bridge septa. Yellow arrow indicates incrassation of the bridging septa in the perinephric space accompanied by increasing thickness of the anterior and posterior fascia. The red arrow indicates incrassation in the bridging septa, thickness of the anterior and posterior renal fascia, and fluid accumulation in the perinephric space.

Inspired by Farrell *et al*. [9], the manifestations of perinephric changes in MDCT images are divided into four grades, which are displayed in Fig. (**2**):

Grade 0: compared with the unaffected side, the perirenal adipose space in the obstruction side is clear, and the density does not increase (Fig. **2A**).

Grade I: fat density increased in the perinephric space of the affected kidney, accompanied by the boundary appearing blurred with linear shadows (Fig. **2B**).

Grade II: in the obstruction side, there was an incrassation of the bridging septa in the ipsilateral perinephric space accompanied by thickening of the anterior and posterior fascia (Fig. **2C**).

Grade III: in the obstruction side, there was an incrassation of the bridging septa in the perinephric space and thickening of the anterior and posterior renal fascia accompanied by fluid accumulation (Fig. **2D**).

### 2.4 Statistical Analysis

Statistical analysis was performed by employing Statistical Package for the Social Sciences software version 25.0 (IBM SPSS, Inc., Chicago, IL). Categorical data were reported as frequency (percentage), and comparisons across groups were performed with the Pearson’s chi-square test or Fisher’s tests. Quantitative data were tested for normality with the Shapiro-Wilk test and described as the mean ± standard deviation if normally distributed or as the median (interquartile range) if not. Intergroup comparisons for continuous variables were performed with one-way ANOVA analysis or Kruskal‒Wallis test based on their normality. The Bonferroni method was used for pairwise intergroup comparisons. Ordinary logistic regression analysis was employed to evaluate the association between serum Cr levels or WBC counts and the grades of perinephric changes. Differences with a *P* value < 0.05 were considered statistically significant.

## 3 RESULTS

In total, 376 cases with AUUTO were included in this study. Among these patients, there were 304 men and 72 women with ages ranging from 16 to 76 years old. According to the secondary perinephric changes observed on the MDCT scans, 376 cases were classified into four grades. Among all cases, 77 (20.5%), 103 (27.4%), 140 (37.2%), and 56 (14.9%) were grade 0, I, II, and III, respectively. There was a significant difference in age among different groups (*P* = 0.009). The average age in the MDCT groups 0, I, II, III was 37 (IQR, 31-44), 39 (IQR, 33-47), 41 (IQR, 33-48) and 43 (IQR, 36-54), respectively. Pairwise intergroup comparisons showed that the age in the MDCT group III was significantly higher than that in the MDCT group 0 (*P* = 0.006).

### 3.1 Location of the Ureteral Calculus

The numbers of patients with calculi located in various ureteral positions were compared across the four MDCT groups. Twenty (26.0%) patients in group 0, 26 (25.2%) patients in group I, 38 (27.1%) patients in group II and 8 (14.3%) patients in group III had a ureteral calculus in the upper ureter and pelvis, while the numbers of patients who had a ureteral calculus in the middle and lower ureter in the MDCT groups 0, I, II, and III were 57 (74.0%), 77 (74.8%), 102 (72.9%) and 48 (85.7%), respectively. This means that most of the ureteral calculi occurred in the middle and lower ureter. There was no statistically significant difference in the locations of the ureteral calculus across the MDCT grade groups (Table **1** *χ^2^* = 3.812, *P* = 0.283).

### 3.2 Grade of Hydronephrosis

The grade of hydronephrosis in different MDCT grade groups was compared. In the MDCT grade 0 group, 20 (26.0%) patients had normal kidneys, 55 (71.4%) patients had mild hydronephrosis, and 2 (2.6%) patients had moderate and severe hydronephrosis. In the MDCT grade I group, 10 (9.7%) patients had normal kidneys, 83 (80.6%) patients had mild hydronephrosis, and 10 (9.7%) patients had moderate and severe hydronephrosis. In the MDCT grade II group, 11 (7.9%) patients had normal kidneys, 118 (84.2%) patients had mild hydronephrosis, and 11 (7.9%) patients had moderate and severe hydronephrosis. In the MDCT grade III group, 3 (5.4%) patients had normal kidneys, 46 (82.1%) patients had mild hydronephrosis, and 7 (12.5%) patients had moderate and severe hydronephrosis. The proportion of patients without hydronephrosis decreased as the MDCT grades increased, whereas the proportions of patients with moderate and severe hydronephrosis increased as the MDCT grades increased. A significant difference was observed in the grade of hydronephrosis among the MDCT grade groups (Table **2**, *P* = 0.002).

### 3.3 Serum Cr and WBC Count Comparison among MDCT Grades

The mean serum Cr levels and WBC counts in each MDCT grade group were compared, as shown in Fig. (**3**). The mean values of the serum Cr levels from MDCT grades 0 to III were 80.0 (IQR, 66.5-90.0) μmol/L, 84.0 (IQR,74.0-101.0) μmol/L, 93.0 (IQR, 78.3-114.0) μmol/L, and 91.0 (IQR,80.3-111.3) μmol/L, respectively. The serum Cr levels were significantly different among the MDCT grade groups (*P* < 0.001), demonstrating that the serum Cr levels increased as the MDCT grades increased. The pairwise intergroup comparisons showed that the serum Cr levels were significantly higher in the MDCT group II and the MDCT group III than those in the MDCT group 0 (*P* < 0.001). The mean WBC counts from MDCT grades 0 to III were 9.26 (IQR, 6.49-11.24) ×10^9^/L, 11.00 (IQR, 8.00-13.00) ×10^9^/L, 11.00 (IQR, 9.00-13.00) ×10^9^/L, and 12.00 (IQR, 10.00-13.75) ×10^9^/L, respectively. Significant differences in terms of WBC counts were observed among the MDCT grade groups (*P* < 0.001), indicating that the WBC count increased as the MDCT grades increased. The pairwise intergroup comparisons showed that the WBC count was significantly higher in the MDCT group II and the MDCT group III than that in the MDCT group 0 (*P* < 0.001).

### 3.4 Association Analysis between MDCT Grades and Serum Cr Levels or WBC Count Examination

Since there were four MDCT grade groups, ordinary logistic regression was performed to investigate the association between MDCT grading and the serum Cr levels and WBC counts. The univariate analysis showed that serum Cr levels (OR: 1.013, 95% CI: 1.006-1.019; *P* < 0.001) and WBC counts (OR: 1.175, 95% CI: 1.109-1.246; *P* < 0.001) were positively associated with MDCT grades (Table **3**).

The test of parallel lines indicated that the proportional odds assumption was upheld (*χ^2^* = 4.357, *P* = 0.360). The multivariate logistic regression analyses showed that WBC counts (OR: 1.171, 95% CI: 1.104-1.241; *P* < 0.001) and serum Cr levels (OR: 1.012, 95% CI: 1.005-1.018; *P* = 0.001) were predictive factors of MDCT grades (Table **3**).

## 4 DISCUSSION

In the present study, perinephric changes secondary to AUUTO were classified into 4 grades based on MDCT images. We found that there was a correlation between the MDCT grades and hydronephrosis, indicating that perinephric changes were closely related to the degree of obstruction and kidney function. Moreover, we found that WBC count and serum Cr levels were predictive factors of MDCT grades.

MDCT, which is simple and safe, is used for the early detection of AUUTO with high sensitivity and specificity. Perinephric changes secondary to AUUTO can be distinctly displayed in MDCT images. Therefore, MDCT is an appropriate method for the rapid diagnosis of acute ureteral obstruction, especially for identifying the location of the calculi and grading of the hydronephrosis [15].

The perinephric space is an area inside the anterior fascia and the posterior fascia, containing the perirenal fat, ureter, adrenal glands, lymphatics, and gonadal vessels [19]. The manifestations of perinephric space changes secondary to acute upper urinary tract obstruction in MDCT images include the thickness and swelling of the renal bridge septum, perinephric fat stranding, the thickness of the anterior and posterior renal fascia, perinephric oedema [20], and perirenal fluid [20-22]. In this study, 79.5% of patients had thickening of the bridging septa, and 52.1% of patients had thickening of the perinephric fascia in the perinephric space of the ipsilateral kidney, which suggested that during acute ureteral obstruction, thickening of the bridging septa was the most common sign of perinephric changes, followed by thickening of the perinephric fascia. As the disease progresses, fluid may accumulate in the perinephric space [21]. In acute ureteral obstruction, the pressure of the collecting system in the ipsilateral kidney increases rapidly and is transmitted to the renal tubule and glomerulus through the collecting ducts. High pressure on the proximal tubule reduces the GFR and glomerular blood flow [13]. If the glomerular filtration pressure exceeds the afferent arteriole pressure, the glomerular filtration will cease, and urine formation stops [13]. The “safety valve” in the kidney will then be opened, and urinary reflux will occur to reduce the pressure on the collecting system through pyelosinus, pyelotubular, pyelolymphatic and pyelovenous pathways [9, 21]. The refluxed urine diffuses to the renal interstitium and enters the space inside the renal capsule, passes through the bridging septa or breaks through the renal capsule to enter the perinephric space, resulting in perinephric fluid accumulation and perinephric oedema [21]. So, the perinephric changes from MDCT grade 0 to III reflect the severity of urinary reflux and the pressure of the collecting system, which indicates that perinephric changes could be used to evaluate the severity of urinary obstruction.

Of the 376 patients in this study, 88.3% had hydronephrosis, and 80.3% had mild hydronephrosis. Consistent with our study, the reported incidence of hydronephrosis caused by urinary obstruction was 69% to 83% [21]. Under urinary obstruction, the high pressure of the collecting system could reduce GFR, and urinary reflux can reduce the pressure on the collecting system. If the obstruction cannot be treated timely, hydronephrosis cannot be avoided. The severity of hydronephrosis in acute ureteral obstruction mainly depends on the degree of obstruction [13], so the hydronephrosis can reflect the degree of ureteral obstruction [21]. Our study found that hydronephrosis was positively associated with perinephric changes. In this study, as the perinephric changes increased from grade 0 to III, the proportions of patients who had moderate and severe hydronephrosis gradually increased. Since hydronephrosis was related to the s of obstruction, we can conclude that perinephric changes can reflect the severity of obstruction. The more severe the perinephric changes, the more severe the obstruction in the upper urinary tract.

Urinary tract obstruction may result in renal function impairment, and patients with nephrolithiasis have a high risk of renal function decline [13, 23]. Serum Cr level is a common parameter for evaluating renal function. Haley *et al*. [7] demonstrated that the mean serum Cr level increased at the time of the stone event. A similar study also reported that the serum Cr levels increased in patients with recurrent kidney stone disease and that kidney function declined over time [8]. In our study, the serum Cr levels increased as the MDCT grades progressed from grade 0 to III, indicating that the severity of the perinephric changes can reflect the functional impairment of the ipsilateral kidney. Similarly, Farrell *et al*. [9] reported that serum Cr levels significantly increased as the severity of peripheral fat stranding increased among patients with acute obstructing ureterolithiasis and demonstrated that the severity and duration of obstruction influence serum Cr levels. In this study, the elevated serum Cr levels could partially be explained by the acute increase in pressure proximal to the obstruction, which leads to a decrease in the net hydraulic pressure gradient, lowering the GFR [13]. At the same time, the rise in intratubular pressure induces secondary renal vasoconstriction, reducing the glomerular blood flow, which induces an increase in serum Cr levels [13]. Moreover, the high pressure of the renal pelvis induces urinary reflux through the pyelovenous and pyelolymphatic pathways, which also leads to an increase in serum Cr levels [9]. In our study, the serum Cr level significantly increased as the MDCT grade progressed from grade 0 to III, indicating that the MDCT grade can reflect the functional impairment of the ipsilateral kidney.

A critical strength of this study lies in its comprehensive inclusion of obstructions across the entire upper urinary tract, from the renal pelvis to the ureterovesical junction. Contrary to potential misinterpretations, lower ureteral calculi (distal to the sacroiliac joint) constituted 74.0-85.7% of cases across MDCT grades (Table **1**). This confirms that our findings are generalizable to all AUUTO, irrespective of calculus location. The lack of association between calculus position and MDCT grades (*P* = 0.283) suggests that perinephric changes reflect the degree of obstruction and resultant pathophysiological stress rather than anatomical site.

In this study, we found that mean WBC counts progressively increased as the MDCT grade increased from grade 0 to III. In line with our study, the Alleemudder group reported that 80.9% of patients with renal colic caused by a solitary ureteric stone had elevated WBC counts [24]. Consistent with these results, an earlier study also reported that WBC counts increased under conditions of renal colic secondary to ureteral calculi [10]. One reason for the elevated WBC counts may be the inflammation caused by the interaction of ureteral stones and mucosa [10]. Damage to the renal tissues in patients with acute ureteral obstruction can lead to inflammatory reactions. Moreover, the stress and ureteral trauma caused by the renal colic can induce demargination of WBC, which would also increase WBC counts [10, 24]. The increase in WBC counts is associated with the inflammatory response and can reflect the kidney function [11]. A recent study demonstrated that elevated WBC count was a predictor of the odds of kidney function decline, suggesting that elevated WBC counts were positively associated with the decline of kidney function [12]. In this study, the mean WBC counts significantly increased with MDCT grades and were positively associated with perinephric changes, indicating that the severity of perinephric changes secondary to acute urinary tract obstruction can reflect the degree of renal function impairment in the ipsilateral kidney.

## 5 LIMITATIONS

Although the perinephric changes secondary to AUUTO were related to the degree of obstruction, they may also be related to the duration of obstruction [25]. This study was strictly limited to patients with acute ureteral obstruction with onset within two weeks, and more detailed comparisons based on onset time need further investigation. In addition, the ages in patients with MDCT grade 0 were significantly different from those with MDCT grades III in this study, while the effect of age on perinephric changes secondary to acute unilateral ureteral obstruction remains to be further explored. In this study, we found that mean WBC counts progressively increased as the MDCT grade increased from grade 0 to III. However, our analysis did not include a differential count of WBC subsets, which might have provided additional insights into the specific inflammatory responses associated with varying degrees of perinephric changes. Future studies could benefit from including differential WBC analysis to further elucidate the relationship between inflammatory cell types and renal function impairment in patients with AUUTO. A prospective multicentre study incorporating novel biomarkers (*e.g*., urinary NGAL) and advanced imaging modalities (*e.g*., dual-energy CT) is needed to further validate this grading system.

## CONCLUSION

The findings of this study underscore the critical role of MDCT-based grading in evaluating perirenal changes secondary to AUUTO and its clinical relevance in reflecting ipsilateral renal functional impairment. By stratifying perinephric alterations into four distinct grades, we demonstrated a robust correlation between MDCT-documented anatomical changes and biochemical markers of renal dysfunction, including serum Cr and WBC counts. These results not only validate the utility of MDCT as a non-invasive diagnostic tool but also provide novel insights into the pathophysiological interplay between urinary obstruction, inflammatory responses, and renal functional decline. The perinephric changes secondary to AUUTO on MDCT images were strongly associated with the degree of obstruction. In this work, inclusive of calculi spanning the renal pelvis to the ureterovesical junction, demonstrates that MDCT grading is universally applicable across upper urinary tract segments, providing a robust tool for early functional assessment. The manifestations of perinephric changes on MDCT images can be used for the assessment of acute renal function decline. Therefore, MDCT grading may help the early diagnosis of the acute renal function impairment and assess kidney function in patients with urinary stones, avoiding the potential risk of infection and deterioration of renal function. By quantifying obstruction severity and its systemic repercussions, this approach empowers clinicians to prioritize interventions, optimize patient outcomes, and curtail the cascading risks of infection and renal deterioration. As precision medicine advances, integrating multimodal imaging and biomarkers will redefine how we diagnose, monitor and treat disease.

## AUTHORS’ CONTRIBUTIONS

The authors confirm their contribution to the paper as follows: NL and HC designed the study and revised the manuscript. FZ collected data, conducted the analysis, and drafted the manuscript. HY conducted the analysis and wrote the manuscript. YQ, GC and ZL assisted with the study design, data collection and manuscript preparation. YD assisted with data acquisition. All authors revised the manuscript and ensured its intellectual content. All authors reviewed and approved the final manuscript. All authors agreed to be accountable for all aspects of the work.

## Figures and Tables

**Fig. (3):**
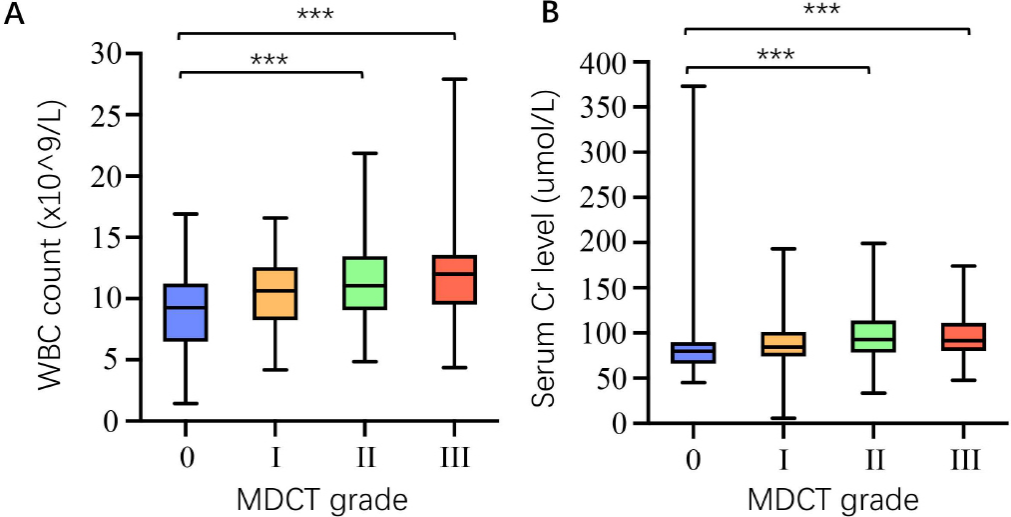
Comparison of WBC count and serum Cr levels (y-axis) among MDCT grade groups (x-axis). (**A**) The WBC count was significantly higher in the MDCT group II and the MDCT group III than that in the MDCT group 0 (*P* < 0.001). (**B**) The serum Cr levels were significantly higher in the MDCT group II and the MDCT group III than those in the MDCT group 0 (*P* < 0.001).

**Table 1:** Comparison of upper urinary calculus location among MDCT grade groups.

Group (n)	Ureteral calculi in the upper ureter and pelvis, n (%)	Ureteral calculi in the middle and lower ureter, n (%)	χ^2^	p-value
Grade 0 (77)	20 (26.0)	57 (74.0)	3.812	0.283
Grade I (103)	26 (25.2)	77 (74.8)		
Grade II (140)	38 (27.1)	102 (72.9)		
Grade III (56)	8 (14.3)	48 (85.7)		

**Abbreviations:** MDCT: multi detector computed tomography.

**Table 2:** Comparison of the hydronephrosis grade among MDCT grade groups.

Group (n)	The grade of hydronephrosis			χ^2^	p value
	Normal, n (%)	Mild, n (%)	Moderate and severe, n (%)		
Grade 0 (77)	20 (5.3%)	55 (14.6%)	2 (0.5%)	20.293	0.002
Grade I (103)	10 (2.7%)	83 (22.1%)	10 (2.7%)		
Grade II (140)	11(2.9%)	118 (31.4%)	11 (2.9%)		
Grade III (56)	3 (0.8%)	46 (12.2%)	7 (1.9%)		

**Abbreviations:** MDCT: multi detector computed tomography.

**Table 3:** Univariate and multiple logistic regression analysis for two factors associated with the grade of perinephric changes secondary to AUUTO.

Univariate				Multivariate		
	OR	95% CI	p value	OR	95% CI	p value
Cr level	1.013	1.006-1.019	< 0.001	1.012	1.005-1.018	0.001
WBC count	1.175	1.109-1.246	< 0.001	1.171	1.104-1.241	< 0.001

**Abbreviations**: Cr: creatinine; WBC: white blood cell; MDCT: multi detector computed tomography.

## Data Availability

The datasets supporting our findings are presented in the article. The datasets analysed during the current study are available from the corresponding author [H.C] on reasonable request.

## LIST OF ABBREVIATIONS

AUUTO: Acute unilateral upper urinary tract obstruction
MDCT: Multi detector computed tomography
Cr: Creatinine
WBC: White blood cell
GFR: Glomerular filtration rate

## STANDARDS OF REPORTING

STORBE guidelines were followed.
